# Canadian infants' nutrient intakes from complementary foods during the first year of life

**DOI:** 10.1186/1471-2431-10-43

**Published:** 2010-06-17

**Authors:** James K Friel, Rhona M Hanning, Corinne A Isaak, Daniel Prowse, Angela C Miller

**Affiliations:** 1 Richardson Centre for Functional Foods and Nutraceuticals 203-196 Innovation Drive, University of Manitoba, Winnipeg, MB, R3T 6C5, Canada; 2 Department of Health Studies & Gerontology, University of Waterloo, Ontario N2L 3G1, Canada; 3 Department of Human Nutritional Sciences, University of Manitoba, Winnipeg, Manitoba, R3T 2N2, Canada

## Abstract

**Background:**

Complementary feeding is currently recommended after six months of age, when the nutrients in breast milk alone are no longer adequate to support growth. Few studies have examined macro- and micro-nutrient intakes from complementary foods (CF) only. Our purpose was to assess the sources and nutritional contribution of CF over the first year of life.

**Methods:**

In July 2003, a cross-sectional survey was conducted on a nationally representative sample of mothers with infants aged three to 12 months. The survey was administered evenly across all regions of the country and included a four-day dietary record to assess infants' CF intakes in household (tablespoon) measures (breast milk and formula intakes excluded). Records from 2,663 infants were analyzed for nutrient and CF food intake according to 12 categories. Mean daily intakes for infants at each month of age from CF were pooled and compared to the Dietary Reference Intakes for the respective age range.

**Results:**

At three months of age, 83% of infants were already consuming infant cereals. Fruits and vegetables were among the most common foods consumed by infants at all ages, while meats were least common at all ages except 12 months. Macro- and micro-nutrient intakes from CF generally increased with age. All mean nutrient intakes, except vitamin D and iron, met CF recommendations at seven to 12 months.

**Conclusions:**

Complementary foods were introduced earlier than recommended. Although mean nutrient intakes from CF at six to 12 months appear to be adequate among Canadian infants, further attention to iron and vitamin D intakes and sources may be warranted.

## Background

Over the last decade, recommendations for the introduction of complementary foods (CF) have undergone various revisions. In 2000, the World Health Organization (WHO) released a publication where it was recommended that infants begin receiving complementary foods at four to six months of age (WHO, 2000; WHO/NHD/00.1, WHO/FCH/CAH/00.6 accessed Nov 30, 2009 http://www.who.int/nutrition/publications/infantfeeding/WHO_NHD_00.1/en/index.html[1,2]). Following the WHO global recommendation in 2001 to extend the duration of exclusive breastfeeding to a full six months of age, corresponding recommendations for the introduction of CF at 6 months of age were also adapted (WHO; Complementary feeding: report of the global consultation (2003); WHO Guiding principles for complementary feeding of the breastfed child (2004); WHO Guiding Principles for feeding non-breastfed children 6-24 months of age (2005). Similarly, in 2005 Health Canada also revised recommendations to delay the introduction of CF to six months of age [3].

Complementary foods (CF) are currently defined as any foods or nutritive beverages (in addition to breast milk or infant formula) that provide the supplementary nutrients required to support infant growth [2]. The WHO [1] recommends that CF have a nutritional composition similar to that of breast milk and that they be consumed two to three times per day at six to eight months and three to four times per day at nine to 11 months.

Various organizations [1-3] have established official recommendations to promote exclusive breastfeeding during the first six months of life. Beyond this point, complementary or "solid" foods are required because breast milk no longer provides adequate amounts of specific nutrients, including vitamins, minerals, protein, and carbohydrate [2]. There is concern that the introduction of CF prior to six months of age may displace breast milk and have a negative impact on nutrient intake [2]. Health Canada [3] supports this concern by stating that both early *and *late introduction of CF (before two to three months and after six months, respectively) are associated with more health risks than benefits.

The purpose of this study was to determine Canadian infants' food group and nutrient intakes from CF (all foods and beverages other than breast milk or formula) during the first year of life. To our knowledge, few studies have assessed total nutrient intakes of infants from CF in North America. Previously, only one study in the United States (US), the Feeding Infants and Toddlers (FIT) Study [4], aimed to determine the national feeding patterns of infants, based on 24-hour parental recalls of the intakes of 3,000 infants and toddlers, aged four to 24 months on phone interview, with second recalls for about 700 participants. The current study is the first of its type and size to be conducted in Canada. Moreover, the four-day recalls specifically examined CF intakes, independently of formula or breast milk. Mean daily nutrient intakes were compared with CF recommendations (Dietary Reference Intakes [DRI], given as Adequate Intakes [AI]) for macro- and micro-nutrients [5-7]. In this article we report nutrient intakes of Canadian infants during the first year of life.

## Methods

In July 2003, -prior to the recommendation by Health Canada to begin complementary feeding at six months of age (3) - Heinz Canada commissioned an independent firm (LLS/DVR Market Research, Toronto, Canada) to conduct a cross-sectional market survey on a nationally representative sample of mothers with infants aged three to 12 months [8]. Subsequently, Heinz agreed to share this information in order to provide representative data on the feeding patterns (summarized previously by Friel et al [9]) and nutrient intakes of Canadian infants. Since this was a market survey generated to understand Canadian consumer habits, no ethical approval was sought for the use of the secondary data. A fifteen dollar honorarium was provided to participants as an incentive.

**Figure 1:**
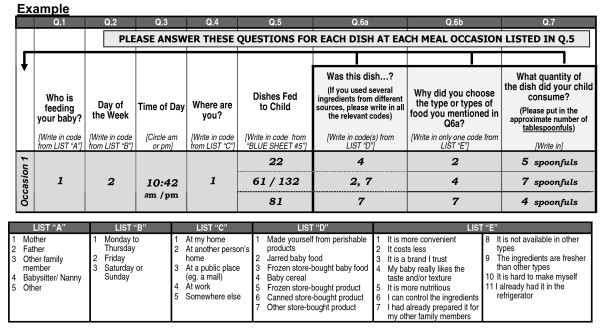
**Dietary diary questionnaire sent to all participants**.

The survey consisted of two components: 1) a questionnaire to assess demographic information and consumer attitudes towards different infant foods; and 2) a self-reported four-day food diary (Figure 1), for which a coded food list of 197 infant and table foods was provided. Respondents were asked to record every item that they fed their youngest child for four days (two weekdays and two weekend days). Each day was divided into four feeding occasions which were classified by who was feeding the infant, time of day, and where the feeding occurred. Each of the feeding occasions was permitted up to five "dishes". A "dish" could be composed of any number of different foods. Each dish was recorded by quantity served (tablespoons) and classified by source (jarred, cereal or homemade). Data were collected for a total of over 77,000 dishes and were organized into 12 categories [9]. For the purpose of our study, all data on human milk and formula consumption was excluded from analysis. The survey did not assess consumption of dietary supplements.

Using the density of each food item, taken from the 2005 Canadian Nutrient File (CNF) [10] or manually measured (weighed by volume in triplicate), the tablespoon data for each food was converted into grams consumed per day. The nutrient content of each food was calculated using the CNF [10]. The final daily CF nutrient intakes for each infant included all foods and beverages listed in their diet record, except formula and human milk.

The mean daily nutrient intakes from CF of each infant was averaged across the days of recording and data from infants of the same age were pooled in SPSSx Version 16 (SPSS Inc., Chicago, IL) to calculate an overall mean daily intake for each month of age (from three to 12 months). To compare the intake from CF to recommended daily nutrient intakes, we calculated the mean intake of each nutrient over the age ranges of one to six and seven to 12 months of age, as used by the DRI committee [5-7]. Within these ranges, the subjects were not evenly distributed across each month of age. Therefore, the mean intakes for each age range (Tables 1, 2 and 3) were weighted to represent the proportion of study subjects at each month of age. All subjects were included in all mean intake calculations, regardless of whether or not they consumed CF.

**Table 1:** Comparison of mean (SD) daily intakes and Dietary Reference Intakes (DRI) for macronutrients

Age (months)	n	Energy (kcal)	Protein (g)	Fat (g)	Carbohydrate (g)
3	110	112 (125)	4 (6)	3 (5)	18 (18)
4	252	118 (148)	3 (5)	2.(4)	22 (28)
5	305	151 (146)	5 (6)	3 (4)	28 (27)
6	396	197 (152)	6 (6)	3 (4)	37 (29)
7	320	233 (145)	8 (6)	5 (5)	42(25)
8	335	290 (194)	10 (8)	6 (6)	50 (34)
9	298	315 (172)	12(8)	7 (6)	53 (29)
10	331	397 (218)	16 (10)	11 (9)	61(32)
11	233	395 (263)	17(12)	12 (11)	57 (35)
12	36	461 (340)	21 (15)	16(14)	61 (47)

DRI for 0-6 m^1^		507	9	31	60
Mean Actual Daily Intake from CF (3-6 m)		144.5	5	3	26
% CF Contributed to DRI^1 ^at 3-6 m		29	50	9	43
DRI for 7-12 m^1^		671	14	30	95
Recommended Amount from CF only (7-12 m)^2^		281	7	6	51
Mean Actual Daily Intake from CF (7-12 m)		326 (222)	13(10)	8 (8)	52 (34)
% CF Contributed to DRI^1 ^at 7-12 m		49	87	28	55
% of CF Recommendation (7-12 m)^2^		116	176	146	103

^1 ^DRI (AI) - includes intake from formula/breast milk and CF [6]

^2 ^Explicitly stated by Institute of Medicine as part of DRI [6]

**Table 2:** Comparison of mean (SD) daily intakes and Dietary Reference Intakes (DRI) for minerals

Age (months)	n	Ca (mg)	Fe (mg)	K (mg)	Na (mg)	Zn (mg)	Mg (mg)	P (mg)	Cu (mg)	Mn (mg)	Se (μg)
3	110	169 (204)	3.6 (4.8)	179 (287)	57 (138)	1 (3)	16 (24)	157 (188)	0.1 (0.1)	0.2 (0.3)	3 (7)
4	252	155 (225)	4.4 (6.9)	188 (356)	46 (111)	1 (8)	16 (29)	154 (223)	0.1 (0.1)	0.3 (0.4)	4 (7)
5	305	184(192)	5.2 (5.6)	284 (339)	76 (114)	2 (8)	24 (28)	186 (193)	0.1 (0.1)	0.4 (0.6)	6 (9)
6	396	211 (197)	6.3 (5.9)	397 (365)	118 (129)	4 (8)	34 (29)	228 (200)	0.2 (0.1)	0.6 (0.6)	7(9)
7	320	241(221)	7.0 (6.0)	459 (326)	153 (140)	5 (9)	40 (35)	262 (220)	0.2 (0.1)	0.7 (0.7)	9 (10)
8	335	271 (238)	7.3 (7.0)	546 (388)	222 (201)	5 (11)	49 (35)	300 (240)	0.2 (0.1)	0.8 (0.6)	11 (11)
9	298	283 (211)	7.1 (5.6)	576 (360)	255 (210)	5 (9)	53 (34)	314 (202)	0.2 (0.1)	0.8 (0.7)	13 (11)
10	331	360 (255)	6.5 (4.8)	703 (437)	372 (267)	5 (9)	66 (38)	387 (240)	0.2 (0.1)	0.9 (0.7)	18 (14)
11	233	370 (324)	5.7 (5.5)	662 (471)	412 (319)	4 (7)	65 (45)	392 (304)	0.2 (0.2)	0.9 (0.8)	19 (16)
12	36	428 (312)	5.2 (4.6)	764 (574)	488 (408)	4 (4)	89 (72)	452 (314)	0.2 (0.2)	1.3 (1.3)	27 (21)

DRI for 0-6 m^1^		210	0.27	400	120	2	30	100	0.2	0.003	15
Mean Actual Daily Intake from CF (3-6 m)		180	4.9	262	74.1	2	23	181	0.1	0.4	5
% CF Contributed to DRI^1 ^at 3-6 m		86	1806	65	62	99	75	181	50	12569	33
DRI for 7-12 m^1^		270	11	700	370	3	75	275	0.22	0.6	20
Recommended Amount from CF only (7-12 m)^2^		140	7.7	440	290	2	55	200	0.04	0.597	11
Mean Actual Daily Intake from CF (7-12 m)		304 (260)	6.7 (5.6)	590 (426)	281 (258)	5 (8)	55 (43)	330 (253)	0.2 (0.1)	0.8 (0.8)	14 (14)
% CF Contributed to DRI^1 ^at 7-12 m		113	61	84	76	164	73	120	91	136	71
% of CF recommendation (7-12 m)		217	87	134	97	273	100	165	500	137	128

^1 ^DRI (AI) - includes intake from formula/breast milk and CF [5,7]

^2 ^For Ca, P, and Mg, explicitly stated by Institute of Medicine as part of total DRI (from both CF and breast milk/formula) [5]; For all other nutrients, calculated by subtracting amount consumed from breast milk (based on concentration in milk [11] and average volume consumed per day [5]) from the total DRI

**Table 3:** Comparison of mean daily intakes and Dietary Reference Intakes (DRI) for vitamins

Age (months)	n	Vit C (mg)	Thiamin (mg)	Riboflavin (mg)	Niacin (mg)	Folate (μg)	Vit B6 (mg)	Vit B12 (μg)	Vit D (μg)	Pantothenic Acid (mg)
3	110	4 (8)	0.2 (0.2)	0.3 (0.3)	2 (4)	11 (19)	0.1 (0.1)	0.1 (0.3)	0.5 (1.0)	0.3 (0.5)
4	252	7 (16)	0.3 (0.3)	0.3 (0.4)	3 (5)	14 (27)	0.1 (0.2)	0.1 (0.2)	0.3 (0.8)	0.2 (0.5)
5	305	12 (24)	0.3 (0.3)	0.4 (0.3)	4 (5)	22 (29)	0.2 (0.2)	0.5 (2.9)	0.3 (0.9)	0.4 (0.5)
6	396	17 (21)	0.4 (0.3)	0.4 (0.3)	5 (5)	30 (26)	0.2 (0.2)	0.6 (4.8)	0.4 (0.8)	0.5 (0.5)
7	320	19 (19)	0.4 (0.3)	0.5 (0.4)	6 (6)	34 (26)	0.3 (0.2)	0.6 (4.6)	0.5 (0.9)	0.6 (0.5)
8	335	23 (21)	0.4 (0.4)	0.5 (0.4)	7 (6)	41 (30)	0.3 (0.2)	1.7 (10.4)	0.6 (1.0)	0.8 (0.6)
9	298	26 (22)	0.4 (0.3)	0.5 (0.3)	7 (5)	44 (28)	0.3 (0.2)	1.0 (4.6)	0.6 (0.9)	0.8 (0.5)
10	331	30 (29)	0.5 (0.3)	0.6 (0.4)	8 (5)	56 (35)	0.4 (0.2)	1.1 (3.7)	1.2 (1.6)	1.1 (0.8)
11	233	26 (24)	0.4 (0.3)	0.6 (0.5)	8 (6)	54 (37)	0.4 (0.2)	1.2 (3.0)	1.3 (1.7)	1.0 (0.8)
12	36	29 (30)	0.4 (0.3)	0.6 (0.5)	9 (6)	59 (44)	0.3 (0.2)	2.8 (10.8)	1.7 (2.1)	1.3 (1.1)

DRI for 0-6 m^1^		40	0.2	0.3	2	65	0.1	0.4	5	1.7
Mean Actual Daily Intake from CF (3-6 m)		9.9	0.3	0.4	4	19	0.1	0.3	0.4	0.3
% CF Contributed to DRI^1 ^at 3-6 m		25	149	120	177	29	136	82	7	20
DRI for 7-12 m^1^		50	0.3	0.4	4	80	0.3	0.5	5	1.8
Recommended Amount from CF only (7-12 m)^2^		22	0.2	0.1	3	14	0.2	0.5	4.7	0.5
Mean Actual Daily Intake from CF (7-12 m)		25 (24)	0.4 (0.3)	0.6 (0.4)	7 (6)	46 (33)	0.3 (0.2)	1.2 (6.2)	0.8 (1.4)	0.9 (0.7)
% CF Contributed to DRI^1 ^at 7-12 m		50	145	140	174	57	108	230	17	48
% of CF Recommendation (7-12 m)		113	217	560	240	326	163	230	18	171

^1 ^DRI (AI) - includes intake from formula/breast milk and CF [5,7]

^2 ^For vit D, explicitly stated by Institute of Medicine as part of DRI [5]; For all other nutrients, calculated by subtracting amount consumed from breast milk (based on concentration in milk [11] and average volume consumed per day [5]) from the DRI

Mean intakes from CF at three to six months were only compared with the total DRI values for zero to six months (intakes from breast milk/formula) as the Institute of Medicine [5-7] does not recommend complementary feeding prior to six months of age. Mean intakes at seven to 12 months of age were compared to official CF recommendations [5-7]. For the macronutrients (energy, protein, fat, and carbohydrate) (Table 1) and select micronutrients (vitamin D, calcium, phosphorus, and magnesium) (Tables 2 and 3), the DRIs explicitly state the proportion required from CF [5,6]. When this amount was not provided in the DRI, it was calculated manually by assuming a daily intake of 0.60 L of breast milk at seven to 12 months of age [5] and the average nutrient composition of breast milk during this stage of lactation [11]. The 12 food groups used in this study are reported in Table 4.

**Table 4:** Proportion of infants consuming different types of complementary foods at each month of age

Type of complementary food	Proportion (%)^1 ^by age (months)								
	3 (*n *= 110)	4 (*n *= 252)	5 (*n *= 305)	6 (*n *= 396)	7 (*n *= 320)	8 (*n *= 335)	9 (*n *= 298)	10 (*n *= 331)	11/12 (*n *= 269)
Infant cereal - All single-grain^2^	83 ^a^	83^a^	89^a^	81^a^	73^b^	74^b^	57^c^	50^c^	40^c^
- Rice	72^a^	74^a^	71^a^	57^a^	45^b^	46^b^	37^c^	30^c^	27^c^
- Mixed	9^c^	16^c^	23^c^	44^b^	59^a^	61^a^	62^a^	56^a^	47^b^
- Barley	12^b^	13^b^	17^a^	20^a^	13^b^	11^b^	9^b^	6^c^	3^c^
- Oatmeal	14^c^	19^c^	33^b^	38^a^	37^a^	35^a^	29^b^	23^c^	18^c^
- Single-grain with fruit	2^c^	3^c^	10^c^	15^b^	16^b^	25^a^	19^b^	22^a^	15^b^
- Mixed-grain with fruit	1^c^	3^c^	7^c^	15^c^	28^a^	35^a^	37^a^	34^a^	26^b^
Toddler foods	1^c^	2^c^	0^c^	1^c^	1^c^	3^b^	5^b^	6^b^	13^a^
Fruit	25^c^	35^c^	47^c^	76^b^	93^a^	94^a^	96^a^	95^a^	91^a^
Vegetables	26^c^	33^c^	61^c^	88^a^	92^a^	92^a^	90^a^	90^a^	90^a^
Meat^3^	3^c^	6^c^	3^c^	15^c^	23^c^	33^b^	44^a^	47^a^	62^a^
Poultry^4^	4^c^	3^c^	6^c^	17^c^	28^b^	35^a^	41^a^	47^a^	53^a^
Fish	1^c^	0^c^	1^c^	1^c^	4^c^	6^b^	12^a^	16^a^	22^a^
Dairy^5^	12^c^	6^c^	7^c^	16^c^	27^c^	40^b^	56^a^	70^a^	76^a^
Beverages^6^	10^c^	12^c^	9^c^	16^c^	24^c^	34^b^	39^a^	51^a^	62^a^
Desserts^7^	5^c^	4^c^	8^c^	21^c^	38^b^	48^a^	50^a^	61^a^	65^a^
Breads/grains^8^	5^c^	4^c^	3^c^	12^c^	25^c^	44^a^	57^a^	69^a^	82^a^
Combination dishes or casseroles^9^	5^c^	3^c^	6^c^	19^c^	39^b^	53^a^	60^a^	72^a^	72^a^

^a^Values in each row with different superscript letters are significantly different (P < 0.05)

^1 ^Percentage of records with at least one item from respective food category

^2 ^Rice, oatmeal, and barley cereals

^3 ^Beef, lamb, ham, pork, veal, and hamburger

^4 ^Chicken and turkey products

^5 ^Eggs, soy- and cow-based milk products; Does not include breast milk or formula

^6 ^Juice, water, and other drinks

^7 ^Pudding, Jello, cookies, candy, apple sauce

^8 ^Pasta, rice, muffins, bread, regular cereals (e.g. Cheerios)

^9 ^Pasta, rice, and/or vegetables with meat, poultry, or fish; mixed vegetables; mixed meat/poultry/fish; macaroni and cheese

Mean intakes for individual food groups at each age were analyzed by one-way ANOVA using SPSSx Version 16 (SPSS Inc., Chicago, IL) to determine the order and prevalence of solid food introduction. Significance was assigned to P < 0.05.

## Results

### Demographics

A total of 14,000 surveys (in English) were mailed to new mothers, selected from the regularly updated Growing Family List [8] - parental information gathered through in-hospital sampling and prenatal education programs. The response rate was 21%, providing data for a total of 2,951 infants. After deleting all subjects with incomplete dietary records (less than one full day), 2,663 infants remained. Of these records, 91% included four days of feeding data. The proportion of responses in each region was 6.6% in the Atlantic Provinces, 36% in Quebec, 29% in Ontario, and 29% in the Western Provinces.

Fifty-six percent of infants came from single-child families, 31% from two-child families, and 13% from families with three or more children. The general trend was toward a lower proportional representation of families with a greater number of children at home. Sixty percent of the mothers in the study were less than 30 years of age, while 40% were 30 years of age or more. Twenty-three percent of mothers had completed an educational level of high school or less, 38% completed college or equivalent, and 39% were University educated. Combined family incomes per year were < $45,000; $45-60,000; and > $60,000 for 41%, 18%, and 41%, respectively.

### Food Intake

The proportions of infants at each month of age who were consuming any foods from the 12 main food categories are presented in Table 4. By three months of age, most infants (83%) were already consuming infant cereal, primarily rice-based. At least one-quarter of subjects at this age were also receiving vegetables and/or fruits, many (12%) were consuming dairy products other than formula or breast milk, and some (1-4%) were already receiving meat, poultry, and fish products.

Food types across the ages of three to 12 months indicated a significant decline in the prevalence of infant cereal consumption beyond nine months. At seven months, mixed cereal was consumed by a significantly greater proportion of subjects and gradually replaced rice cereal in prevalence. Beginning at eight or nine months and extending throughout the remainder of infancy, there was a significant increase in the proportion of infants receiving meat, poultry, fish, dairy, beverages, desserts, breads, and combination dishes/casseroles.

Across all months of age, fruits and vegetables were among the top four most common food groupings found on all diet records. After nine months of age, greater than 90% of infants were consuming vegetables and/or fruits. On the other hand, meats were generally among the least common foods found on all diet records, with the exception of records for infants aged 12 months. A more detailed description of food consumption data was previously reported [9].

### Nutrient Intakes

Complementary food intake provided an increasing amount of energy and macronutrients from three to 12 months of age (Table 1). Mean daily intakes of energy and macronutrients met the recommendations for CF at seven to 12 months of age [5-7]. Although carbohydrates provided 62% of total daily energy from CF (versus 15% from protein and 23% from fat), mean daily carbohydrate intake from CF did not reach the seven to 12 month recommendation (51 g/d) [6] until nine months. Mean daily fat intake from CF did not meet the recommendation (5.7 g/d) [6] until eight months. This may have contributed to the slightly lower mean energy intake at seven months, relative to CF recommendations [6].

On average, energy intake from CF alone accounted for only 29% of the total daily energy recommendation if contributions from milk are included, at three to six months and 49% of the recommendation at seven to 12 months [6]. By nine months, infants' mean daily energy and macronutrient intakes from CF exceeded the seven to 12 month CF recommendations [6].

Daily mineral intakes from CF at each month of age are reported in Table 2. The mean intakes of iron, zinc, manganese, and phosphorus from CF alone satisfied the total daily recommendations for infants aged three to six months, recognizing that these recommendations were based on breast milk composition and nutrient bioavailability [5,7]. By seven to 12 months of age, all mineral intakes (except iron) satisfied the daily recommendations for CF [5,7] (Table 2). Mean iron intake never met the seven to 12 month recommendation for CF [7]. Mineral intakes tended to increase or remain stable with age, with the exceptions of iron (decreased after nine months) and zinc (decreased gradually after eight months).

Table 3 depicts daily vitamin intakes from CF at each month of age. At three to 12 months, average CF intakes alone provided thiamin, riboflavin, niacin, and vitamin B6 in amounts that exceeded total daily recommendations [5,7]. Vitamin intakes from CF generally increased steadily from three to 12 months of age. On average, daily CF intake alone provided less than one-fifth of the total recommendation for vitamin D (0.8 of 5 μg [5]) at all ages.

## Discussion

Although the response rate was low in this study (21%), respondents were representative of the Canadian population, being similar to the proportion of Canada's total population in each region at that time (Atlantic Provinces, Québec, Ontario, and the Western Provinces; 7.6%, 24%, 38%, and 30%, respectively [12]) and the proportion of Canada's total births in each region; 6%, 22%, 39%, and 31.7%, respectively) [13]. During the same year in which the survey was conducted, the distribution of number of children per family for the Canadian population was 44%, 39%, and 17%, respectively (families with three or more children, two-child families, single-child families) [14], very similar to that reported in this study. Accounting for mothers' age in this study, a representative distribution of Canadian females' (aged 15-44 years) educational backgrounds at the time would have been 55% with high school or less, 26% with college, and 19% with university degrees/diplomas/certificates [15]. In addition, combined annual family incomes in this study were similar to all families in the Canadian population in 2000 (36%, 16%, and 48%, respectively) [16]. Overall, the survey participants convey a representative sample of the Canadian population at the time of the data collection.

Although some studies have measured intakes of CF, most were conducted in developing countries [17-21] or their analyses included only select nutrients (usually energy and protein [22,23]). Only three studies (all conducted in the Unites States) have thoroughly examined infants' nutrient intakes in industrialized countries. Two of these studies focused on energy and protein intakes only.

Dewey [23] provided 'estimated requirements', calculated directly from the DRIs and recommendations of the WHO/FAO, for most of the macro- and micronutrients from CF only. Estimates for energy and protein were indicative of 'usual intakes' of infants in the United States [18,24]. Dewey [23] estimated that CF contributed a mean of 129 kcal and 311 kcal to the total diet from six to eight and nine to 11 months, respectively, as compared to 240 kcal and 369 kcal, respectively, in the current study. Average intakes of protein from CF were estimated to be 1.9 g and 4.0 g at six to eight and nine to 11 months, respectively [23]. In the current study, the actual mean intakes of protein from CF were approximately 8.1 g and 14.7 g, respectively.

In the Darling Study [22] of breastfed (n = 73) and formula-fed (n = 46) American infants aged three to 12 months, no significant difference in energy and protein intakes from solid foods were found between infants fed formula versus breast milk. The mean energy intakes of these two groups were approximately 105 kcal, 282 kcal, and 497 kcal per day at six, nine, and 12 months of age [22]. In the current study, mean energy intakes of infants aged nine (315 kcal/d) and 12 months (461 kcal/d) form CF were within 10% of those found in the Darling Study [22]. At six months, however, the subjects in our study were consuming nearly two-fold greater energy from CF as those in the Darling Study [22]. Finally, the FIT Study in the United States [4,25-27] measured infants' nutrient intakes from all foods and beverages, including human milk, formula, and nutrient supplements. The types of CF provided to infants appear to be similar to the current study [26], with a few exceptions. Juice was second only to infant cereals as a source of CF energy in six to 11 month-old infants in this US study, but was consumed by fewer than half of Canadian infants until 10 months of age. Also, a decline in vegetable intake as children aged was observed in the FIT study [26], but not the current study. The FIT Study [4] revealed that nutrient supplements were given to only ~8% of four to five month old infants and 19% of six to 11 month-old infants [27]. The contribution of supplements to nutrient intake was relatively low, with the exception of vitamin D for which supplements provided 5% of the intake of four to five month-olds and 14% of the intake of those aged six to 11 months.

While we report mean CF intakes for zero to 12 months of age (Tables 2, 3 and 4), the corresponding recommendations [5-7] represent intakes from breast milk or formula only and do not include CF. As a result, only the intakes of subjects aged seven to 12 months were directly compared with DRIs [5-7].

Canadian infants' mean energy, fat, protein, and carbohydrate intakes from CF were adequate (Table 1). In the current study, energy and protein consumption appeared to be higher across all ages when compared to 'usual' intakes [23]. It has been suggested that infants aged six to 11 months require approximately 19 to 43% of the total energy in solid foods as lipids [18]. In the current study, infants in this age range consumed only 16% to 28% of mean energy from CF as fat.

According to the recommended intakes from CF only [5,7], all micronutrient intakes in the current study, except iron and vitamin D, were considered adequate (Tables 2 and 3). Mean daily intakes of iron and vitamin D accounted for 87% and only 18%, respectively, of the recommendation from CF.

### Vitamin D Intakes

The Institute of Medicine [5] states that 2.5 μg/day of vitamin D is required to prevent rickets. However, even at this dietary intake, insufficient sunlight may lead to low serum 25-hydroxyvitamin D [5]. In the current study, mean daily intake of vitamin D from CF was 0.4 μg at zero to six months and 0.8 μg at seven to 12 months. These intakes are well below the recommended amount for the prevention of rickets [5], as well as for overall dietary adequacy [7,9] (Table 4). Although infant formula would be expected to meet the needs of non-breastfed infants, it is noteworthy that vitamin D-deficiency rickets remains an issue among a small number of Canadian infants, affecting 2.9 per 100,000 children aged zero to 18 years in 2007 [28]. Also, eight months has been identified as the mean age for the development of vitamin D deficiency (without rickets) [29].

The low intakes of vitamin D from CF observed in the current study may be due to the fact that many infant foods naturally contain little or no vitamin D and are not fortified. For example, a typical single-grain infant cereal (rice, barley, or oatmeal) does not contain vitamin D. In Canada, only cow's milk and margarine must be fortified with vitamin D [29]. Health Canada recommends that infants not be given cow's milk until nine to 12 months of age [3]. Fatty fish and egg yolks contain a significant amount of vitamin D [29] and are the only natural sources in the Canadian food supply [29]. According to the current data, most infants (78 to 99%) do not consume fish in their first year of life.

### Iron Intakes

The relatively low intake of iron from CF is also a concern as iron stores are depleted by six months of age and the concentration of iron in breast milk is insignificant [11]. Beyond six months of age, the majority of infants' daily iron must be supplied by CF [11]. Mean daily iron intakes from CF were lower than recommended for subjects aged seven to 12 months. Most of the CF consumed by these infants does not contain significant amounts of iron, with the exception of iron-fortified infant foods and red meats. For example, Heinz canned fruits and vegetables provide 2 to 10% of infants' daily value of iron per jar [30], while their canned meat and combination dishes provide no more than 20% of daily iron/jar [31]). Of the most commonly consumed CF (cereals, fruits, and vegetables), only infant cereals contain an appreciable amount of iron (approximately 90-100% of daily value). Intakes of meat, poultry, fish, and dairy (eggs) were not significant until eight to nine months of age (Table 4). The FIT study found that infant cereal was the most important complementary source of all minerals, except sodium and potassium, for infants [26].

Infant foods are fortified with either elemental iron or ferric pyrophosphate, both of which provide iron of low bioavailability [32,33]. Although iron-fortified infant formulas contain highly absorbable iron (ferrous sulfate) [32], not all infants receive formula. Iron status is an issue for Canadian infants with 24% reported to have low iron stores [34]. To ensure infants aged six to 12 months are able to meet their daily iron requirements, fortification with other forms of iron and addition of absorption-enhancing compounds (such as ascorbic acid) may need to be considered.

Health Canada recommends iron-containing foods, including cooked egg yolks, as the first CF [3]. Egg yolks contain 1.3 times more iron per gram than ground beef and one yolk provides 6% and 12% of infants' daily recommended iron and vitamin D, respectively [10]. As a result, egg yolks may be the perfect complement to breast milk and formula [35].

### Timing of Introduction of CF

The data from the current study (Table 4) suggests that many infants are beginning complementary feeding earlier than recommended [5-7,36,37]. In keeping with Canadian tradition [8], the primary first food is infant cereal (Table 4) and parents are not waiting until six months to start solids. The high proportion of infants consuming cereals at three months (83%) implies that they may have been started even earlier. It is noteworthy that in the US FIT study conducted in 2002, two thirds of infants had been introduced to CF by four to six months, though data were not collected before four months [26]. In a sample of infants from Southern Ontario, Canada, 18% had received infant cereal by four months [36]. Kattelmann and colleagues [38] found that the introduction of CF at three or four months rather than six months did not lead to a difference in iron or zinc status later in infancy. Mehta *et al *[39] found no difference in growth or body composition when infants were fed CF earlier than three months. However, neither study included breastfed infants.

### Limitations

The four-day diet records used in this study may not provide an accurate representation of infants' actual energy intakes [40]. However, protein and fat can be calculated accurately based on four days, while two- and three-day records are sufficient for analysis of micronutrient and carbohydrate intakes [40]. Mothers in this study appeared to be better educated than the average Canadian mother [15]. It may be that the more educated mothers self-selected to respond to this survey.

## Conclusions

This study was based on data from a Heinz marketing survey and we did not have control over its design and administration. Based on key demographic indicators, these data appear to represent the infant population from the majority of Canadian provinces. This is the first study to examine nutrient intakes of a national sample of Canadian infants since 1976 [41]. In 2010, Heinz will be repeating their Canadian market survey. From new data, we will determine whether infant feeding patterns and nutrient intakes were influenced by the 2004/2005 national recommendations to extend the duration of exclusive breastfeeding to six months of age [37,42].

In conclusion, intakes of Vitamin D and iron among Canadian infants may be low, although breast milk and formula feeding were not taken into account. Canadian infants appear to be starting the consumption of solid foods early and not in accordance with current recommendations

## Abbreviations

CF: complementary foods; FIT: Feeding Infants and Toddlers; WHO: World Health Organization; DRI: Dietary Reference Intakes; AI: Adequate Intakes; CNF: Canadian Nutrient File; US: United States.

## Competing interests

The authors declare that they have no competing interests.

## Authors' contributions

The authors' responsibilities were as follows - JKF, CAI, AM and RH: writing the manuscript; JKF: design of the study, data collection, and writing the manuscript; DP: database creation and analysis. All authors have read and approved the final manuscript.

## Pre-publication history

The pre-publication history for this paper can be accessed here:

http://www.biomedcentral.com/1471-2431/10/43/prepub
